# Ergothioneine Improves Aerobic Performance Without Any Negative Effect on Early Muscle Recovery Signaling in Response to Acute Exercise

**DOI:** 10.3389/fphys.2022.834597

**Published:** 2022-02-09

**Authors:** Théo Fovet, Corentin Guilhot, Pierre Delobel, Angèle Chopard, Guillaume Py, Thomas Brioche

**Affiliations:** DMEM, INRAE, Montpellier University, Montpellier, France

**Keywords:** ergothioneine, muscle, exercise, antioxidant, exercise performance, exercise recovery

## Abstract

Physical activity is now recognized as an essential element of healthy lifestyles. However, intensive and repeated exercise practice produces a high level of stress that must be managed, particularly oxidative damage and inflammation. Many studies investigated the effect of antioxidants, but reported only few positive effects, or even muscle recovery impairment. Secondary antioxidants are frequently highlighted as a way to optimize these interactions. Ergothioneine is a potential nutritional supplement and a secondary antioxidant that activates the cellular NRF2 pathway, leading to antioxidant response gene activation. Here, we hypothesized that ergothioneine could improve performance during aerobic exercise up to exhaustion and reduce exercise-related stress without impairing early muscle recovery signaling. To test this hypothesis, 5-month-old C56B6J female mice were divided in two groups matched for maximal aerobic speed (MAS): control group (Ctrl; *n* = 9) and group supplemented with 70 mg ergothioneine/kg/day (ET; *n* = 9). After 1 week of supplementation (or not), mice performed a maximum time-to-exhaustion test by running on a treadmill at 70% of their MAS, and gastrocnemius and soleus muscles were collected 2 h after exercise. Time to exhaustion was longer in the ET than Ctrl group (+41.22%, *p* < 0.01). Two hours after exercise, the ET group showed higher activation of protein synthesis and satellite cells, despite their longer effort. Conversely, expression in muscles of metabolic stress and inflammation markers was decreased, as well as oxidative damage markers in the ET group. Moreover, ergothioneine did not seem to impair mitochondrial recovery. These results suggest an important effect of ergothioneine on time-to-exhaustion performance and improved muscle recovery after exercise.

## Introduction

Physical activity is now recognized as an essential element of healthy lifestyles. However, the frequent practice of intensive physical activity produces high stress levels, particularly oxidative stress and inflammation, that must be managed (Wang et al., 2006; Sureda et al., 2009; Kawamura and Muraoka, 2018). Many athletes in aerobic-dominant sports empirically use anti-oxidant supplementation to counteract the overproduction of reactive oxygen and nitric species (RONS) and the inflammation occurring during exercise (Macera et al., 2003; Bassel-Duby and Olson, 2006; Warburton et al., 2006; Schnohr et al., 2015; Ranchordas et al., 2017). These supplementation strategies may have several objectives, such as performance gain, or improving muscle adaptation/recovery after a training session and/or competition (Knapik et al., 2016). RONS overproduction during exercise can affect performance, especially in long endurance events, exhaustive exercise, or extended and repeated high intensity exercise bouts (McKenna et al., 2006; Paschalis et al., 2016; Reid, 2016). Indeed, RONS overproduction, especially by mitochondria, NADPH and xanthine oxidase systems, during exercise can induce muscle strength loss and fatigue (Powers and Jackson, 2008; Powers et al., 2011, 2016). This is explained by the many effects of oxidizing molecules, leading to protein, lipid and DNA damage, and also by their implication in different pathways, for instance, muscle protein synthesis, protein degradation, excitation-contraction coupling (calcium movements) and apoptosis (Powers et al., 2011, 2016).

Several groups have investigated the effect of antioxidants as a strategy to reduce exercise-related damage (Braakhuis and Hopkins, 2015; Ranchordas et al., 2017). However, they found only few positive effects, and these results are debated. Some studies, mainly using N-acetylcysteine (a glutathione precursor), reported a performance increase or fatigue delay during aerobic exercise (e.g., cycling or running time to exhaustion; Medved et al., 2004; Braakhuis and Hopkins, 2015; Rhodes and Braakhuis, 2017; Paschalis et al., 2018). However, antioxidant use in training is currently not recommended, despite the fact that they can increase performance during aerobic exercise. Indeed, RONS are essential components of the muscular adaptations associated with exercise (Margaritelis et al., 2020). During aerobic exercise, the transient RONS overproduction stimulates PGC1α (peroxisome proliferator-activated receptor gamma coactivator 1-alpha) production in skeletal muscle cells, the major regulator of mitochondrial biogenesis and metabolism adaptation (Pilegaard et al., 2003; Lin et al., 2005) through induction of specific genes, such as NRF-1 (nuclear respiratory factor 1) and mtTFA (mitochondrial transcription factor A; Pilegaard et al., 2003; Lin et al., 2005; Silveira et al., 2006; Kang et al., 2009; Gomez-Cabrera et al., 2015). Some studies have shown that primary antioxidants, such as vitamin C and E, may prevent the activation of the PGC1α pathway, thus blunting mitochondrial adaptations to exercise and consequently reducing the gain in maximal oxygen uptake and maximal endurance time (Gomez-Cabrera et al., 2008; Ristow et al., 2009; Strobel et al., 2011; Merry and Ristow, 2016). Moreover, other studies using a resistance training protocol and primary antioxidant supplementation highlighted the inhibition of some training adaptation (e.g., muscle power increase, muscle fatigue delay, and muscle hypertrophy). This inhibition seems to act through a lower increase in insulin sensitivity that normally stimulates the activation of the mTOR signaling pathway (Malm et al., 1997; Wang and Proud, 2006; Schiaffino et al., 2013; Paulsen et al., 2014a,b; Bjørnsen et al., 2016). For example, after a 10-week strength training protocol in women, the gains in peak torque and total work were lower in the group with vitamin C and E supplementation than in the placebo group (Dutra et al., 2018). In addition, Arc-Chagnaud et al. showed that during chronic inactivity, RONS are necessary to maintain muscle function (Arc-Chagnaud et al., 2020). They also found that expression of the main components of the PGC1α and mTOR pathways were decreased in the group with antioxidant supplementation during the re-loading phase, in agreement with the results by Gomez-Cabrera et al. on adaptation to exercise. These findings highlight again the importance of the interactions between redox balance, physical exercise/mechanical constraints, and antioxidant effects (Reid, 2001, 2016; Schnohr et al., 2015).

These interactions might be optimized by personalizing the supplement cocktail (Paschalis et al., 2016; Margaritelis et al., 2018a,b), or by developing new secondary antioxidants that interact with the antioxidant response elements (ARE) of genes rather than activate RONS scavengers (Merry and Ristow, 2016). Secondary antioxidants (e.g., resveratrol) show beneficial effects on exercise performance and oxidative stress (Wu et al., 2013; Baltaci et al., 2016). However, due to their low bioavailability, their utilization in humans is limited and other molecules must be tested/developed (Walle et al., 2004). Ergothioneine, a secondary antioxidant derived from fungi and bacteria, is a potential candidate (Tang et al., 2018). Although ergothioneine is not produced by mammals, it can be found at high concentrations in human and animal tissues through the action of a specific transporter (OCTN1; Halliwell et al., 2016). This molecule is a nuclear factor erythroid 2-related factor 2 (NRF2) pathway activator and allows the activation of genes implicated in the cell antioxidant response (Ma, 2013; Hseu et al., 2015; Crilly et al., 2016). Several studies have described its antioxidant and anti-inflammatory properties *in vitro* and *in vivo* (Cheah and Halliwell, 2012; Halliwell et al., 2016). For example, ergothioneine protects several cell types against oxidative damage and apoptosis after exposure to RONS or UV radiation (Aruoma et al., 1999; Markova et al., 2009). Moreover, administration of pure ergothioneine in middle-aged healthy individuals decreases oxidative stress and inflammation markers (Cheah et al., 2017).

The objectives of this study were to test ergothioneine effects on the aerobic performance and to analyze its impact on muscle molecular adaptations to exercise, which are normally blunted by primary antioxidants. We hypothesized that pure ergothioneine does not impair the early adaptations to aerobic exercise and improves performance in a time-to-exhaustion treadmill protocol.

## Materials and Methods

### Ethical Review and Study Design

**Figure 1:**
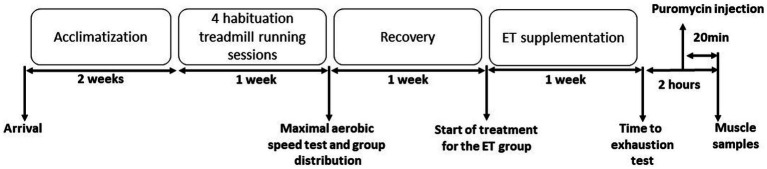
Experimental protocol. Mice were acclimatized to their cages for 2 weeks, before undergoing four treadmill running habituation sessions. One week before supplementation initiation, the maximal aerobic speed (MAS) was measured by running on the treadmill. Then, mice were distributed in the control (Ctrl) and ergothioneine (ET) supplementation group according to their MAS. After 1 week of ET supplementation (or not for the Ctrl group), mice performed a time to exhaustion test at 70% of their individual MAS. Puromycin was injected 100 min after the test end and muscles were collected after sacrifice at 120 min after the exercise end.

This study was approved by the Languedoc-Roussillon ethics committee (APAFIS#28764–-2020122115407491). Five-month-old C56B6J female mice were acclimated and assigned to two groups: control group (Ctrl; *n* = 9) and group supplemented with ergothioneine (ET; *n* = 9). After four habituations sessions to treadmill exercise, the maximal aerobic speed (MAS) on the treadmill was measured 1 week before supplementation initiation. Mice in the two groups were MAS-matched. After 1 week of supplementation with pure ergothioneine, mice performed a double-blind time-to-exhaustion exercise on the treadmill at 70% of their individual MAS. Then, mice were sacrificed 2 h after the exercise end, and muscle samples were collected. Puromycin (40 nmol/g of body weight) was injected with intraperitoneal injection (i.p) 20 min before euthanasia (Merle et al., 2019; Figure 1).

### Supplementation

Pure ergothioneine was provided by Tetrahedron (Paris, France). Ergothioneine was given at the concentration of 70 mg/kg/day for 1 week, as previously described (Tang et al., 2018). Ergothioneine was diluted in the drinking water (each mouse was in one cage) and adjusted according to variations in water intake and body weight.

### Maximal Aerobic Speed Measurement

MAS was measured on a treadmill (Exer-6 M Treadmill; Columbus instruments, Oh, United States) using a standard protocol (Gouraud et al., 2019). Mice were progressively acclimatized to treadmill running by increasing the speed and duration of exercise (four habituation sessions). The MAS measurement protocol began with a warming up time (5 min at 6 m·min^−1^, 2 min at 8 m·min^−1^, and 2 min at 10 m·min^−1^). Then, the treadmill running velocity was increased by 2 m·min^−1^ every min until exhaustion, defined as the inability to start running again after 10 s. The speed at exhaustion was considered to be the MAS.

### Time-to-Exhaustion Test

The time-to-exhaustion test was performed on the treadmill. After the warming up step (5 min at 6 m·min^−1^ and 5 min at 10 m·min^−1^), running speed was increased by 2 m·min^−1^ every minute until 70% of each mouse MAS. Then, mice run at this speed until exhaustion. Exhaustion was defined as the inability to return to treadmill running after 10 s.

### Muscle Samples

*Soleus* and *gastrocnemius* muscles were collected straight after sacrifice. Samples were rapidly frozen in liquid nitrogen and stored at −80°C for mRNA and protein extraction.

### Western Blot Analysis

Muscles (*n* = 1 gastrocnemius and *n* = 2 soleus/mouse) were homogenized in 10 volumes of lysis buffer [50 mM Tris–HCl (pH 7.5), 150 mM NaCl, 1 mM egtazic acid, 1 mM EDTA, 100 mM NaF, 5 mM Na_3_VO_4_, 1% Triton X-100, 1% sodium dodecyl sulphate (SDS), 40 mM β-glycerophosphate, and a protease inhibitor mixture (P8340; Sigma-Aldrich, Saint-Louis, MO, United States)] and centrifuged at 10,000 × *g* for 10 min (4°C). Fifty micrograms of each protein extract were loaded on stain-free 4–20% precast gels (4568095; Bio-Rad, Hercules, CA, United States) for protein separation by electrophoresis followed by transfer to nitrocellulose membranes (Trans-Blot Turbo Blotting System; Bio-Rad). Membranes were blocked with 50 mM Tris–HCl (pH 7.5), 150 mM NaCl, and 0.1% Tween-20 containing 5% skim milk or bovine serum albumin and incubated at 4°C with primary antibodies overnight (Table 1). Membranes were then incubated with the relevant secondary antibodies at room temperature for 1 h (Table 1), followed by the Pierce ECL kit (32106; Thermo Fisher Scientific, Waltham, MA, United States). Bands were visualized with the ChemiDoc Touch Imaging System (Bio-Rad) and quantified with Image Lab™ Touch (version 5.2.1). The Stain-Free technology was used as loading control (Gilda and Gomes, 2015; Vigelsø et al., 2015; Faden et al., 2016; Pagano et al., 2018).

**Table 1:** Antibodies used for Western Blotting.

Antibody	Reference	Manufacturer	Dilution
Phosphorylated 4EBP1	9451S	Cell signaling	1:1,000
4EBP1	9644S	Cell signaling	1:1,000
4-HNE	46,545	Abcam	1:2,000
Phosphorylated AMPKα	50,081	Cell signaling	1:1,000
AMPKα	5,832	Cell signaling	1:1,000
Phosphorylated p38	9,211	Cell signaling	1:1,000
P38	9,212	Cell signaling	1:1,000
Phosphorylated GSK3	9,331	Cell signaling	1:1,000
GSK3	5,676	Cell signaling	1:1,000
SOD1	40,163	Genetex	1:1,000
SOD2	116,093	Genetex	1:1,000
Phosphorylated p53	12,571 T	Cell signaling	1:1,000
P53	2524S	Cell signaling	1:1,000
Phosphorylated RPS6	5364S	Cell signaling	1:1,000
RPS6	3944S	Cell signaling	1:1,000
Ubiquitin	Sc-8,017	Santa-Cruz	1:200
MAFbx	Sc-33,782	Santa-Cruz	1:200
MurF1	Sc-27,642	Santa-Cruz	1:200
P62	ab56416	Abcam	1:1,000
LC3	L7543	Sigma	1:400
Phosphorylated ULK1	6,888	Cell signaling	1:1,000
ULK1	8054S	Cell signaling	1:1,000
Cytochrome C	Sc-13,560	Santa-Cruz	1:200
PGC1-α	AB3242	Millipore	1:1,000
COX IV	Sc-69,360	Santa-Cruz	1:200
TNF-α	Sc-52,746	Santa-Cruz	1:200
IL-1β	Sc-7,884	Santa-Cruz	1:200
PAX7	Sc-81,975	Santa-Cruz	1:200
MyoD	Sc-304	Santa-Cruz	1:200
Myogenin	Sc-398,002	Santa-Cruz	1:200
Myf-5	Sc-302	Santa-Cruz	1:200
Spry-1	13013S	Cell signaling	1:1,000
Puromycin	MABE343	Millipore	1:2,000
Anti-mouse HRP	7,076	Cell signaling	1:5,000
Anti-rabbit HRP	7,074	Cell signaling	1:5,000
Anti-goat HRP	Sc-2,953	Santa-Cruz	1:4,000

### Carbonylated Proteins

The Oxyblot Protein Oxidation Detection Kit (Millipore, United States) was used for carbonylated protein analysis. Protein samples were denatured with 12% SDS (final concentration: 6% SDS). Samples were then derivatized by adding 2,4-dinitrophenylhydrazine (DNPH) and incubated at room temperature for 15 min. The reaction was stopped by adding a neutralization solution before electrophoretic separation on 4–20% precast gels (5678094; Bio-Rad) followed by transfer onto nitrocellulose membranes (Bio-Rad; Trans-Blot Turbo Blotting System). Membranes were washed in PBS-T and incubated with the primary antibody diluted (1:150) in blocking solution at room temperature for 1 h. Membranes were then washed in PBS-T and incubated with a secondary antibody diluted in blocking solution (1:300) at room temperature for 1 h. Then, membranes were washed and proteins were visualized with the ChemiDoc Touch Imaging System after incubation with the Pierce ECL kit (32106; Thermo Scientific) for 5 min. Carbonylated proteins were quantified with Image Lab™ Touch (version 5.2.1) relative to Ponceau Red staining (loading control).

### Gastrocnemius Muscle RNA Extraction and Reverse Transcription

RNA could be extracted only from *gastrocnemius* samples because both *soleus* muscles were used for protein analysis. Each muscle sample was crushed in 1 ml of TRIzol in a FastPrep-24 device and incubated at room temperature for 5 min. This was followed by addition of 0.2 ml chloroform and incubation at room temperature for 3 min. Tubes were centrifuged at 9,384 *g*, 4°C for 15 min. The upper phase was collected and incubated at room temperature with 0.5 ml of isopropanol for 1 h, followed by centrifugation at 9,384 *g*, 4°C, for 5 min. Pellets were washed twice with 75% ethanol followed by centrifugation at 7,500 *g* for 5 min. RNA pellets were resuspended in 30 μl of RNA-free water and RNA quantified with a spectrophotometer (260 nm). After reverse transcription of each RNA sample using the High-Capacity cDNA Reverse Transcription Kit (4368813, Applied Biosystem), samples were stored at −20°C.

### Quantitative PCR Analysis

Quantitative PCR (qPCR) was performed with the SensiFAST SYBR Hi-ROX Kit (Bioline) and the primers are listed in Table 2 using a StepOnePlus Real-Time PCR system. Data were analyzed with the StepOnePlus 2.3 software. The relative mRNA levels were normalized to the levels of the *Rps9* and tubulin housekeeping genes that were unaffected by the experimental protocol. Results were expressed using the comparative cycle threshold. The relative changes in the level of a specific gene were calculated with the ΔΔCT formula.

**Table 2:** Primers used for the qPCR assays.

Target gene	Forward	Reverse
*Rps9*	CGGCCCGGGAGCTGTTGACG	CTGCTTGCGGACCCTAATGT
Tubulin	CTGGAACCCACGGTCATC	GTGGCCACGAGCATAGTTATT
*4ebp1*	GGTGAGTTCCGACACTCCAT	GGGGACTACAGCACCACTCC
*Akt1*	ACCCAGCAGTATGCCAAGTC	GGAAGTCGCTGGTATTGAGC
*AMPKα*	CCTTCGGGAAAGTGAAGGT	GAATCTTCTGCCGGTTGAGT
*Atg7*	TGGCGTTTAGCCCAGATTG	AGGTTCACCATCCTCGG
*Gpx1*	GGTTCGAGCCCAATTTTACA	CCCACCAGGAACTTCTCAAA
*Gsk3b*	AACTGACTTCCTGTGGCCTG	GCAGCCTTCAGCTTTTGGTA
*IGF1*	AGCAGCCTTCCAACTCAATTAT	GAAGACGACATGATGTGTATCTTTATC
*Il1β*	AGTTGACGGACCCCAAAAG	AGCTGGATGCTCTCATCAGG
*Il-6*	TGGTACTCCAGAAGACCAGAGG	AACGATGATGCACTTGCAGA
*MAFbx*	AGTGAGGACCGGCTACTGTG	GATCAAACGCTTGCGAATCT
*Mtor*	CTGCAGCGTGGGGTTTAG	GTGGGATCATGCAGGTGTACT
*MurF1*	TCCTGCAGAGTGACCAAGG	GGCGTAGAGGGTGTCAAAC
*MyoD*	AGCACTACAGTGGCGACTCA	GGCCGCTGTAATCCATCAT
*Myogenin*	ACAGGCCTTGCTCAGCTC	CGCTGTGGGAGTTGCATT
*Nrf1*	GGTGGGGGACAGATAGTCCT	ATGCTCACAGGGATCTGGAC
*Nrf2*	CCGCTACACCGACTACGATT	ACCTTCATCACCAACCCAAG
Parkin	GCCCGGTGACCATGATAG	GTGTCAGAATCGACCTCCACT
*Pax7*	GTCGGGTTCTGATTCCACAT	GCGAGAAGAAAGCCAAACAC
*Pgc1α*	GGAGCCGTGACCACTGACA	TGGTTTGCTGCATGGTTCTG
*Pink1*	GCGAAGCCATCTTAAGCAAA	TGGGACCATCTCTGGATCTT
*Redd1*	CCAGAGAAGAGGGCCTTGA	CCATCCAGGTATGAGGAGTCTT
*Rps6*	CTTGAGGAGCTCAAACTGGG	CTGGACTTCAGCCATCCAAG
*SOD1*	AAAATGAGGTCCTGCACTGG	ACCATCCACTTCGAGCAGAA
*SOD2*	GCTTGATAGCCTCCAGCAAC	AACTCAGGTCGCTCTTCAGC
*Tfam*	AGGGAGCTACCAGAAGCAGA	TGACTTGGAGTTAGCTGCTCTTT
*Tnfα*	CTGTAGCCCACGTCGTAGC	TTTGAGATCCATGCCGTTG

### Statistical Analyses

All data are expressed as the mean ± SD and the significance level was set at *p* < 0.05. Differences between groups were evaluated using the *t*-test or the Mann–Whitney U test when the data deviated from the normal distribution. For weight comparison, two-way ANOVA for paired data was used. Statistical analyses were done with the Statistica software 7.1, and graphs were generated with GraphPad Prism4 (San Diego, United States).

## Results

### Mouse Body Weight

The mean body weight was not different between groups (Ctrl and ET) throughout the protocol: 23.66 ± 1.90 g and 23.7 ± 2.08 g before the 1-week supplementation and 23.56 ± 1.34 g and 23.52 ± 1.61 g after the 1-week supplementation in the Ctrl and ET group, respectively.

### Maximal Aerobic Speed and Time to Exhaustion

**Figure 2:**
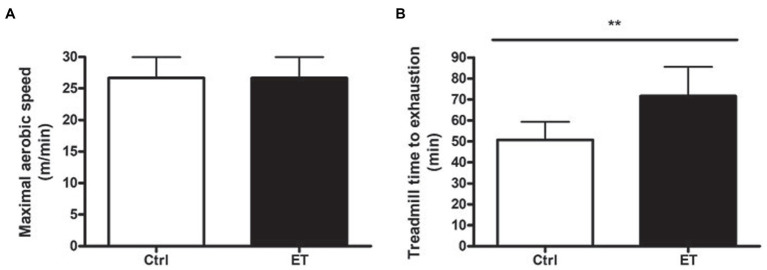
Functional tests. **(A)** Maximal aerobic speed (treadmill running; m/min) in the ergothioneine (ET; *n* = 9) and control (Ctrl; *n* = 9) groups before starting ergothioneine supplementation. **(B)** Time to exhaustion test (min) by running on a treadmill at 70% of the maximal aerobic speed after 1 week of ergothioneine supplementation (ET) or not (Ctrl). *p < 0.05, **p < 0.01 vs. Ctrl.

MAS was exactly the same in the Ctrl and ET group at baseline (26.66 ± 3.32 m/min). Conversely, after 1 week of supplementation, time to exhaustion at 70% of MAS was significantly higher in the ET than Ctrl group: 71.55 ± 14 min and 50.4 ± 8.41 min (+41,22%; *p* < 0.01; Figure 2).

### Muscle Protein Synthesis and Regulating Pathway

**Figure 3:**
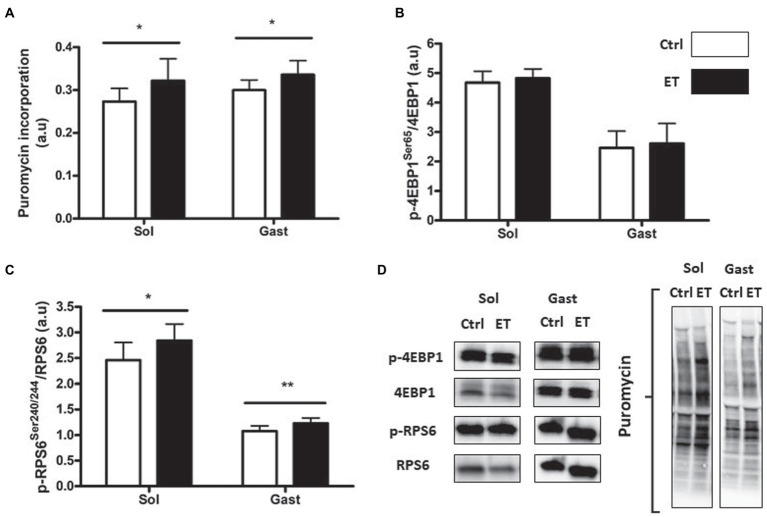
Ergothioneine effect on protein synthesis markers at 2 h post-exercise. Evaluation of puromycin incorporation and protein synthesis marker expression in *soleus* (*n* = 2/mouse) and *gastrocnemius* (*n* = 1/mouse) samples. **(A)** Quantification of protein synthesis by measuring puromycin incorporation in muscles. **(B)** Phosphorylated 4EBP1 (Ser65)/total 4EBP1 protein ratio post-exercise. **(C)** Phosphorylated RPS6 (Ser240/244)/total RPS6 protein ratio. **(D)** Representative Western Blots. *p < 0.05; ** *p* < 0.01 vs. Ctrl.

**Figure 4:**
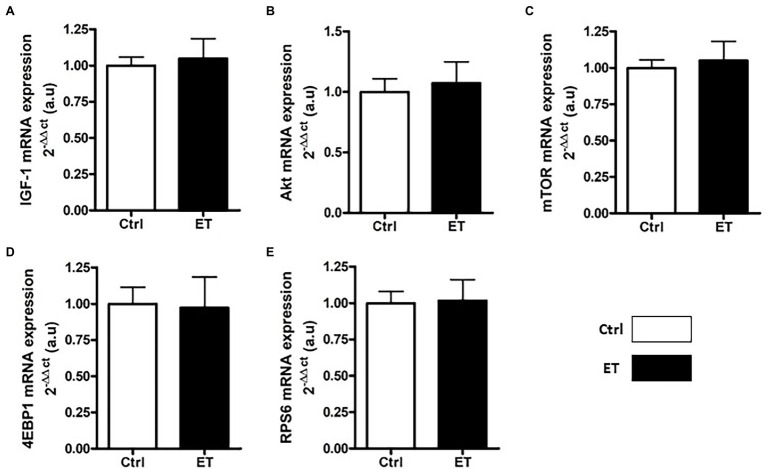
Ergothioneine effect on transcription of protein synthesis markers. Expression of genes encoding protein synthesis markers in *gastrocnemius* samples (*n* = 1/mouse). **(A)** IGF1 mRNA level. **(B)** Akt mRNA level. **(C)** Mtor mRNA level. **(D)** 4ebp1 mRNA level. **(E)** Rps6 mRNA level; *p < 0.05 vs. Ctrl.

After 1 week of supplementation, puromycin incorporation (as a marker of global protein synthesis) in *gastrocnemius* and *soleus* was higher in the ET than Ctrl group (+12% and + 17.8%, respectively; *p* < 0.05 for both) and RPS6 phosphorylation (+14.1%, *p* < 0.01; and + 15.7%, p < 0.05, respectively; Figure 3). 4EBP1 phosphorylation showed no difference between groups and muscles. *Akt*, *Mtor*, *Rps6*, *4ebp1*, and *IGF1* mRNA levels in *gastrocnemius* were not different (Figure 4).

### Muscle Protein Breakdown Markers

**Figure 5:**
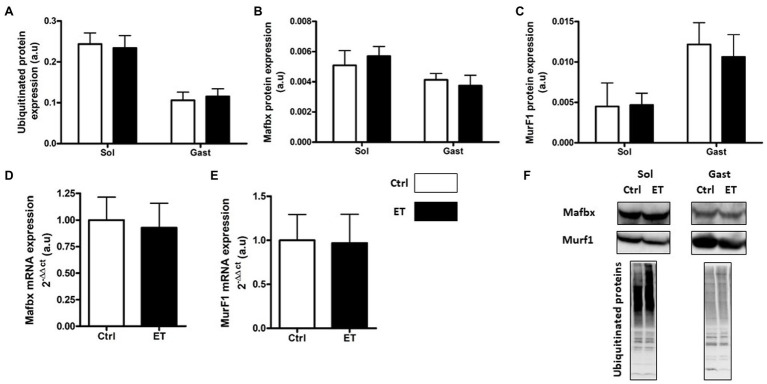
Ergothioneine effect on ubiquitin proteasome markers (UPS) 2 h after exercise. UPS markers in *soleus* and *gastrocnemius* samples and expression of genes encoding UPS markers in *gastrocnemius* samples. **(A)** Total ubiquitinated proteins. **(B)** MAFbx protein expression. **(C)** MurF1 protein expression. **(D)** Mafbx mRNA expression. **(E)** MurF1 mRNA expression. **(F)** Representative Western Blots; *p < 0.05 vs. Ctrl.

**Figure 6:**
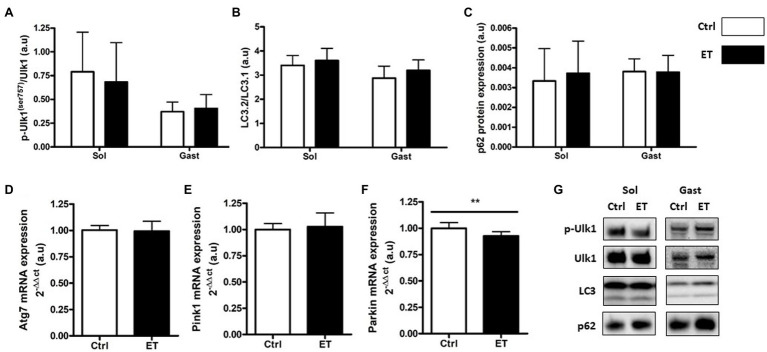
Ergothioneine effect on autophagy markers 2 h after exercise. Autophagy markers in *soleus* and *gastrocnemius* muscle and expression of genes encoding autophagy markers in *gastrocnemius* muscle. **(A)** Phosphorylated ULK1 (Ser757)/total ULK1 ratio. **(B)** LC3.2/LC3.1 protein ratio. **(C)** p62 protein expression. **(D)** Atg7 mRNA level. **(E)** Pink1 mRNA level. **(F)** Parkin mRNA level. **(G)** Representative Western Blots; **p < 0.01 vs. Ctrl.

Two hours after the time-to-exhaustion-test, the muscle expression (gene and protein) of the E3 ligase MurF1 and MAFbx, markers of the Ubiquitin Proteasome System (UPS), was comparable in Ctrl and ET mice (Figure 5), as well as total ubiquitinated protein content. Moreover, ULK1 phosphorylated at Ser757, LC3.2/LC3.1 expression ratio, and p62 protein level, three autophagy markers, were comparable in *gastrocnemius* and *soleus* samples from both groups (Figure 6). Analysis of two mitochondrial autophagy markers showed that *Parkin* mRNA level in *gastrocnemius* was lower in the ET group than Ctrl group (−8%; *p* < 0.01), whereas *Pink1* expression was comparable between groups (Figure 6).

### Metabolic Stress Markers

**Figure 7:**
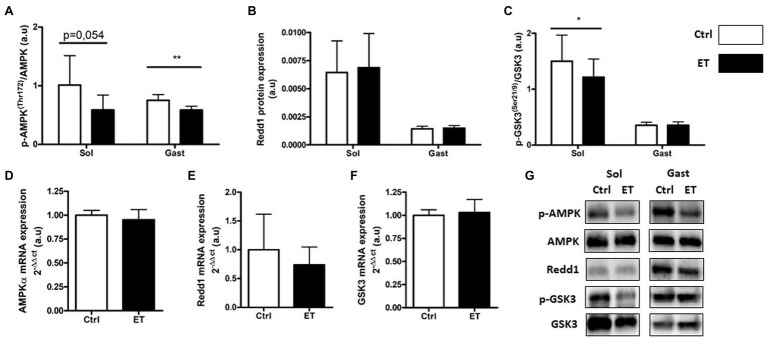
Ergothioneine effect on metabolic stress markers 2 h after exercise. Metabolic stress markers in *soleus* and *gastrocnemius* muscle and expression of genes encoding metabolic markers in *gastrocnemius* muscle. **(A)** Phosphorylated AMPKa (Thr172)/total AMPKa ratio. **(B)** Redd1 protein expression. **(C)** Phosphorylated GSK3 (Ser21/9)/total GSK3 ratio. **(D)** AMPKa mRNA level. **(E)** Redd1 mRNA level. **(F)** Gsk3 mRNA level. **(G)** Representative Western Blots; *p < 0.05; **p <0.01 vs. Ctrl.

In the ET group, AMPKα phosphorylation was significantly lower in *gastrocnemius* samples (−22.05%; *p* < 0.01 vs. Ctrl group) but not in *soleus* samples (−41.9%; *p* = 0.054; Figure 7). Conversely, the expression of its gene *AMPKα* was comparable in ET and Ctrl *gastrocnemius* samples. Redd1 protein and mRNA expression levels were similar between groups. GSK3 phosphorylation was comparable between groups in *gastrocnemius*, but was reduced in *soleus* samples from ET mice (−30.45%; *p* < 0.05 vs. Ctrl). *Gsk3* mRNA levels in *gastrocnemius* samples were similar between groups.

### Inflammation Markers

**Figure 8:**
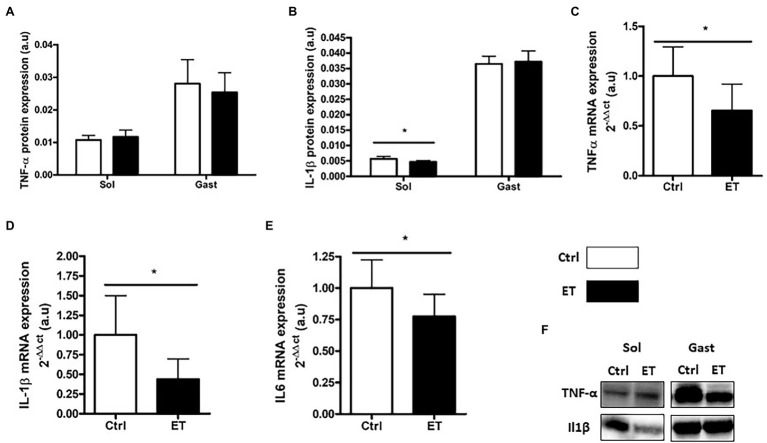
Ergothioneine effect on muscle inflammatory markers 2 h after exercise. Inflammatory marker (protein and gene) expression in *soleus* and *gastrocnemius* samples. **(A)** TNF-a protein expression. **(B)** IL-1b protein expression. **(C)** Tnfa mRNA level. **(D)** IL-1b mRNA level. **(E)** IL6 mRNA level. **(F)** Representative Western Blots; *p < 0.05 vs. Ctrl.

TNF-α protein expression in *gastrocnemius* and *soleus* samples was comparable between groups (Figure 8). Conversely, *Tnf-α* mRNA level in *gastrocnemius* samples was significantly lower in the ET group (−34%; p < 0.05). IL-1β protein expression was lower in ET than Ctrl *soleus* samples (−16.4%; p < 0.05), but not in *gastrocnemius* samples. *Il1β* and *Il6* mRNA levels were decreased in ET *gastrocnemius* samples (−56%, *p* < 0.01; and − 22%, *p* < 0.05 vs. Ctrl samples, respectively).

### Oxidative Stress Markers

**Figure 9:**
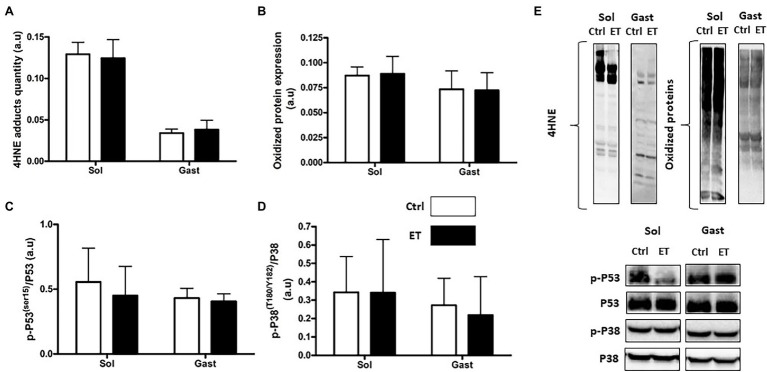
Ergothioneine effect on oxidative stress markers 2 h after exercise. Oxidative stress markers in *soleus* and *gastrocnemius* samples. **(A)** 4HNE adduct quantification. **(B)** Oxidized protein expression. **(C)** Phosphorylated p53 (Ser15)/total p53 ratio. **(D)** Phosphorylated p38(Thr180/Tyr182)/p38 ratio. **(E)** Representative Western Blots; *p < 0.05 vs. Ctrl.

Lipid (4HNE adduct quantification) and protein peroxidation (oxidized protein expression) in *gastrocnemius* and in *soleus* samples were comparable between groups (Figure 9). The p53 phosphorylated on Ser15/total p53 and the p38 phosphorylated on Thr180/Tyr182/total p38 ratios were similar in *gastrocnemius* and *soleus* samples from both groups.

### Antioxidant Cell Defenses

**Figure 10:**
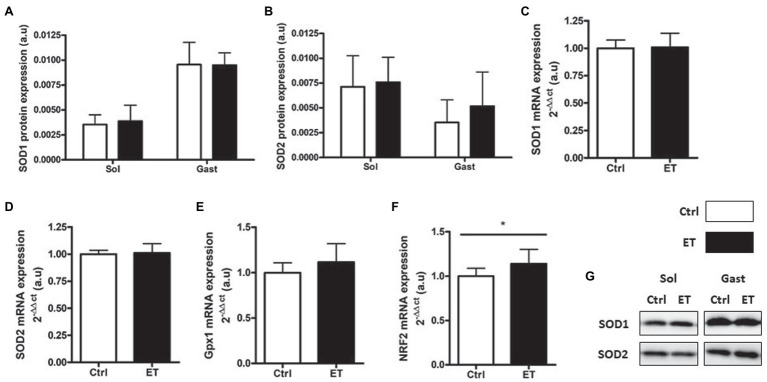
Ergothioneine effect on antioxidant defense markers 2 h after exercise. Antioxidant defense markers in *soleus* and *gastrocnemius* samples. **(A)** SOD1 protein expression. **(B)** SOD2 protein expression. **(C)** SOD1 mRNA level. **(D)** SOD2 mRNA level. **(E)** Gpx1 mRNA level. **(F)** Nrf2 mRNA level. **(G)** Representative Western Blots; *p < 0.05 vs. Ctrl.

SOD1 and SOD2 protein expression in *gastrocnemius* and in *soleus* showed no difference between groups (Figure 10) as well as *SOD1*, *SOD2* and *Gpx1* mRNA levels in *gastrocnemius*. Conversely, *Nrf2* mRNA level was significantly higher in the ET group (+14%; *p* < 0.05 vs. Ctrl).

### Mitochondrial Pathway

**Figure 11:**
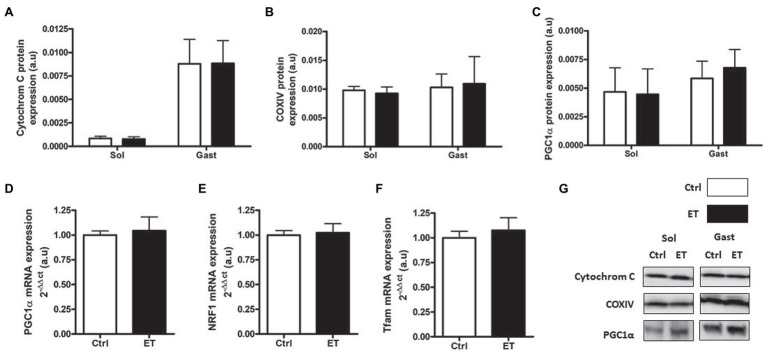
Ergothioneine effect on mitochondrial markers 2 h after exercise. Mitochondrial markers. **(A)** Cytochrome C protein expression. **(B)** COX IV protein expression. **(C)** PGC1a protein expression. **(D)** PGC1a mRNA level. **(E)** NRF1 mRNA level. **(F)** Tfam mRNA level. **(G)** Representative Western Blots; *p < 0.05 vs. Ctrl.

PGC1α, cytochrome C and COX IV protein content were similar in ET and Ctrl *gastrocnemius* and *soleus* samples (Figure 11) as well as *Tfam*, *Pgc1*α and *Nrf1* mRNA levels in *gastrocnemius*.

### Muscle Satellite Cells

**Figure 12:**
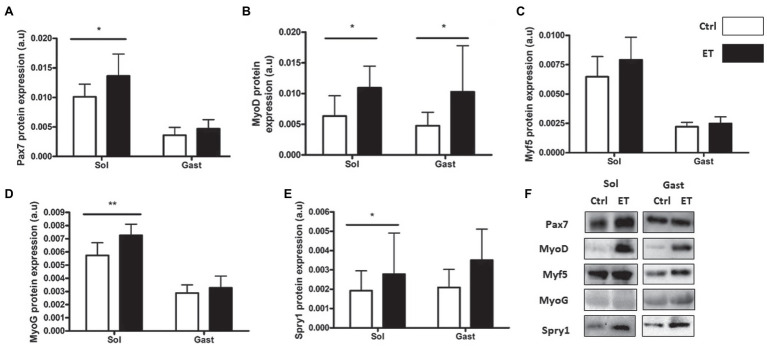
Ergothioneine effect on satellite cell markers 2 h after exercise. Satellite cell markers in *soleus* and *gastrocnemius* samples. **(A)** PAX7 protein expression. **(B)** MyoD protein expression. **(C)** Myf-5 protein expression. **(D)** Myogenin protein expression. **(E)** Spry-1 protein expression. **(F)** Representative Western Blots; *p < 0.05; **p < 0.01 vs. Ctrl.

**Figure 13:**
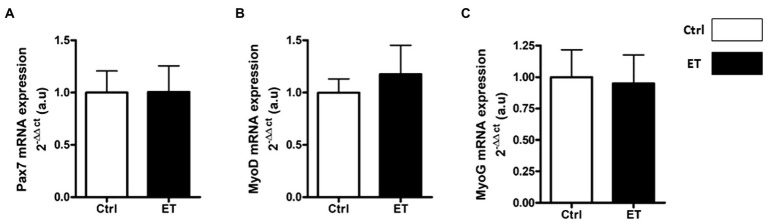
Ergothioneine effect on transcription regulation of satellite cell markers. Satellite cell marker transcription regulation in *gastrocnemius* muscle. **(A)** Pax7 mRNA level. **(B)** MyoD mRNA level. **(C)** Myogenin mRNA level. *p < 0.05 vs. Ctrl.

PAX7, MyoD, Myogenin and Spy-1 protein expression levels were all increased in ET *soleus* (+35.1%, *p* < 0.05; +72.8%, *p* < 0.05; +26.6%, *p* < 0.01; +67,4%, *p* < 0.05 vs. Ctrl, respectively), but only MyoD in *gastrocnemius* (+116.2%, *p* < 0.05 vs. Ctrl; Figure 12). *Pax7*, *MyoD* and *Myogenin* mRNA levels in *gastrocnemius* were similar between groups (Figure 13).

## Discussion

This study evaluated the effect of 1 week of ergothioneine supplementation on time to exhaustion (treadmill running) and on the profile of early markers of muscle recovery after exercise.

At baseline, MAS was identical in Ctrl and ET mice (26.66 ± 3.32 m/min). After 1 week of supplementation, time to exhaustion was 41.22% longer in the ET than Ctrl group (tested at 70% of the individual MAS), thus supporting our hypothesis that ergothioneine might increase performance in exhaustive aerobic exercise. This result is similar to what reported using antioxidant molecules, such as N-acetylcysteine (McKenna et al., 2006), resveratrol (Wu et al., 2013), and natural food extracts that contain antioxidants (Huang et al., 2012; Wang et al., 2012).

Following exhaustive exercise, the expression of oxidative stress and oxidative damage markers in muscle increases in function of the exercise intensity (Merle et al., 2019) and duration (Vezzoli et al., 2016; Thirupathi et al., 2021). In our study, exercise-induced oxidative damage (4HNE, oxidized proteins and p53 phosphorylation on Ser15) in muscle was similar in ET and Ctrl mice, despite the longer intense effort performed by ET animals that should have led to higher RONS production (Fisher-Wellman and Bloomer, 2009). Similarly, the protein and mRNA levels of factors implicated in the enzymatic antioxidant defenses were similar between groups. However, *Nrf2* mRNA expression was upregulated in *gastrocnemius* in the ET group, in agreement with the study by Hseu et al. (2015) that investigated ergothioneine role as NRF2 stimulator and confirmed a greater REDOX management. However, one of the limitations of our study was the absence of a control group that did not allow determining the extent of exercise-induced RONS production and its time course (Michailidis et al., 2007). On the other hand, exercise-induced inflammatory response markers (*Tnf*α, *Il1β*, and *Il6* mRNA) were lower in the ET than Ctrl group as well as IL-1β protein content in *soleus*. This finding could be explained not only by ergothioneine anti-inflammatory properties (Cheah et al., 2017) but also by a lower metabolic stress induced by exercise (Pagano et al., 2014; Merle et al., 2019), suggesting a better adaptation to exhaustive effort in the ET group. Indeed, the metabolic stressor sensor AMPKα was less activated in the ET than Ctrl group, reflecting a less depleted energy state. GSK3 phosphorylation also was decreased in the ET group, which results in lower glycogen synthase activation (Rayasam et al., 2009). These findings suggest that exercise-induced metabolic stress was less important in the ET group, despite their longer physical effort. More studies are needed to precisely understand ergothioneine role in metabolic stress.

Many studies have investigated the use of antioxidants and anti-inflammatory drugs for muscle recovery after physical exercise and found that they inhibit mitochondrial adaptations (Gomez-Cabrera et al., 2008; Merry and Ristow, 2016). Here, 2 h after exercise, we found that PGC1α protein level (the main mitochondriogenesis marker) was comparable in ET and Ctrl mice as well as *Pgc1*α, *Nrf1*, and *Tfam* mRNA expression. This suggests that ergothioneine did not impair early mitochondrial adaptations, unlike primary antioxidants, such as vitamin C and E (Gomez-Cabrera et al., 2015). The comparable COX IV, cytochrome C and citrate synthase protein levels in muscles between groups indicates that 1 week of ergothioneine supplementation did not affect mitochondrial content and capacity. Therefore, ergothioneine may increase time to exhaustion mainly by regulating REDOX balance and by managing muscle fatigue, rather than by increasing the muscle oxidative capacity. More studies are needed to test this hypothesis.

Several studies reported that during muscle recovery, primary antioxidants slow down the protein synthesis pathway. Two hours after exercise, protein synthesis markers were upregulated in the ET group compared with Ctrl, despite the longer effort of ET mice that might decrease protein synthesis (Merle et al., 2019). This is consistent with the lower metabolic stress observed in ET muscles, because such stress normally delays protein synthesis activation after physical activity (MacDougall et al., 1995; Bolster et al., 2002; Dreyer et al., 2006; Thomson, 2018). This finding also indicates that ergothioneine antioxidant action did not impair early protein synthesis after exercise, unlike primary antioxidants (Pagano et al., 2014; Bjørnsen et al., 2016). Conversely, expression of UPS and autophagy markers, the two main pathways involved in protein degradation after exercise, was comparable between groups. As autophagy and UPS are redox-sensitive pathways, this finding confirms that the REDOX status in the ET group was not impaired despite the longer exercise (Powers et al., 2016). On the other hand, *Parkin* mRNA expression (a mitochondrial degradation marker) was downregulated in the ET group, possibly suggesting a protective effect of ergothioneine against mitochondrial RONS production (Barodia et al., 2017).

Finally, assessment of muscle regeneration through quantification of muscle satellite cell markers showed higher PAX7 and Spry-1 protein levels in the ET group. This may suggest a greater pool of quiescent satellite cells and promotion of their asymmetrical division (Shea et al., 2010; Dumont et al., 2015). MyoD and Myogenin expression levels (two satellite cell activation markers) also were increased in the ET group suggesting a better regeneration activation. As RONS promotes MyoD and Myogenin activation (Anderson, 2000; Moal et al., 2017), this finding may indicate that ergothioneine did not inhibit RONS beneficial effects on muscle regeneration through satellite cell activity. Moreover, ergothioneine intake might favor satellite cell asymmetrical division and thus the maintenance of the satellite cell pool (Troy et al., 2012). In our experimental conditions, our findings suggest that this effect of ergothioneine may be greater in *soleus* muscle through Myogenin upregulation after exercise (Snijders et al., 2015), but more data are needed to validate this hypothesis. MyoD and Myogenin increased expression is also in accordance with NRF2 function in satellite cells (Dai et al., 2020; Kourakis et al., 2020). Indeed, NRF2 is needed to maintain PAX7 and MyoD expression in muscle (Narashimhan et al., 2014) and to promote satellite cell proliferation and differentiation (Murakami and Motohashi, 2015).

## Conclusion

To conclude, ergothioneine supplementation significantly improved time-to-exhaustion in mice. Moreover, the post-exercise inflammatory response and metabolic stress were less important in the ET group despite the longer exercise time. Ergothioneine also slightly improved early protein synthesis and did not impair mitochondrial recovery. Moreover, ergothioneine promoted the quiescent pool maintenance and activation after exercise. These results suggest that ergothioneine could help to better manage exercise-induced muscle damage and recovery. In addition, ergothioneine anti-inflammatory and antioxidant effects could be interesting for limiting muscle deconditioning related to hypoactivity or ageing. Ergothioneine might be useful also for the management of diseases in which inflammatory and oxidative stress play a major role, such as chronic obstructive pulmonary disease and type 2 diabetes.

## Data Availability Statement

The original contributions presented in the study are included in the article/supplementary material, further inquiries can be directed to the corresponding authors.

## Ethics Statement

The animal study was reviewed and approved by the Languedoc-Roussillon Ethics Committee (APAFIS#28764-2020122115407491).

## Author Contributions

TF: study design, experimentation, analysis, writing, and validation. CG, PD, GP, and AC: experimentation and validation. TB: study design, experimentation, writing, and validation. All authors contributed to the article and approved the submitted version.

## Funding

This study was funded by the French Centre National d’Etudes Spatiales (CNES), 4800000797.

## Conflict of Interest

The authors declare that the research was conducted in the absence of any commercial or financial relationships that could be construed as a potential conflict of interest.

## Publisher’s Note

All claims expressed in this article are solely those of the authors and do not necessarily represent those of their affiliated organizations, or those of the publisher, the editors and the reviewers. Any product that may be evaluated in this article, or claim that may be made by its manufacturer, is not guaranteed or endorsed by the publisher.
